# Skeletal Muscle Fibrosis in the mdx/utrn+/- Mouse Validates Its Suitability as a Murine Model of Duchenne Muscular Dystrophy

**DOI:** 10.1371/journal.pone.0117306

**Published:** 2015-01-21

**Authors:** Kelly M. Gutpell, William T. Hrinivich, Lisa M. Hoffman

**Affiliations:** 1 Imaging Program, Lawson Health Research Institute, Department of Anatomy and Cell Biology, Western University, London, ON, Canada; 2 Department of Medical Biophysics, Western University, London, ON, Canada; 3 Imaging Program, Lawson Health Research Institute, Department of Anatomy and Cell Biology, Western University, Department of Medical Biophysics, Western University, London, ON, Canada; Faculty of Animal Sciences and Food Engineering, University of São Paulo, Pirassununga, SP, Brazil, BRAZIL

## Abstract

Various therapeutic approaches have been studied for the treatment of Duchenne muscular dystrophy (DMD), but none of these approaches have led to significant long-term effects in patients. One reason for this observed inefficacy may be the use of inappropriate animal models for the testing of therapeutic agents. The mdx mouse is the most widely used murine model of DMD, yet it does not model the fibrotic progression observed in patients. Other murine models of DMD are available that lack one or both alleles of utrophin, a functional analog of dystrophin. The aim of this study was to compare fibrosis and myofiber damage in the mdx, mdx/utrn+/- and double knockout (dko) mouse models. We used Masson’s trichrome stain and percentage of centrally-nucleated myofibers as indicators of fibrosis and myofiber regeneration, respectively, to assess disease progression in diaphragm and gastrocnemius muscles harvested from young and aged wild-type, mdx, mdx/utrn+/- and dko mice. Our results indicated that eight week-old gastrocnemius muscles of both mdx/utrn+/- and dko hind limb developed fibrosis whereas age-matched mdx gastrocnemius muscle did not (p = 0.002). The amount of collagen found in the mdx/utrn+/- diaphragm was significantly higher than that found in the corresponding diaphragm muscles of wild-type animals, but not of mdx animals (p = 0.0003). Aged mdx/utrn+/- mice developed fibrosis in both diaphragm and gastrocnemius muscles compared to wild-type controls (p = 0.003). Mdx diaphragm was fibrotic in aged mice as well (p = 0.0235), whereas the gastrocnemius muscle in these animals was not fibrotic. We did not measure a significant difference in collagen staining between wild-type and mdx gastrocnemius muscles. The results of this study support previous reports that the moderately-affected mdx/utrn+/- mouse is a better model of DMD, and we show here that this difference is apparent by 2 months of age.

## Introduction

Treatment strategies for Duchenne muscular dystrophy (DMD), a severe neuromuscular degenerative disorder, have been ongoing for decades with little significant long-term efficacy reported [1]. While scientific and technological advancements have enhanced patient quality of life, the disease remains invariably fatal. The majority of current research into treating DMD involves the restoration of the protein dystrophin, which is absent or non-functional in these patients. Of these, a number of studies in DMD patients have used cell therapy to replace dystrophin, reporting an increase in dystrophin-positive myofibers [2–8]. While these studies have successfully reintroduced the protein to dystrophic skeletal muscle, improvements in function have been limited. A recent study by Hogrel et al reported a potential long-term effect of a myoblast transplant into an affected female carrier of the dystrophin mutation. Although long-term functional effects could not be definitively concluded from this study, the results suggest beneficial effects of cell therapy [9]. To date, the most commonly used murine model to test cell replacement and other strategies in a pre-clinical setting has been the mdx mouse, which lacks dystrophin due to an X-linked mutation in its gene [10, 11]. Although the mdx mouse is a genetic homolog of the human disease, it has been shown that this model does not mimic the pathology observed in patients because up-regulation of utrophin, a dystrophin analogue, partially compensates for the absence of the cytoplasmic protein [12]. Additionally, the longer telomere length present in inbred laboratory mice confer a greater regenerative capacity of muscle progenitor cells in these animals compared to human skeletal muscle [13]. As a result of these differences, the disease does not manifest severely in mdx mice. Specifically, fibrosis, a hallmark feature of DMD in patients, is not observed in mdx mice [14]. A lack of dystrophin in skeletal muscle leads to decreased sarcolemmal integrity, which causes an increase in cell membrane permeability. As a result, an influx of calcium ions causes increased protein degradation that eventually leads to muscle cell death. Inflammatory cells that infiltrate the site of necrosis are a rich source of transforming growth factor beta (TGFβ). TGFβ then exerts its profibrotic effect on fibroblasts, which then increase production of extracellular matrix (ECM) proteins. An excessive amount of ECM production leads to the eventual onset of fibrosis [15]. The fact that DMD patients develop severe fibrosis whereas mdx mice do not may, in part, explain why treatments performed in murine studies have been ineffective in human trials. Fibrosis limits the amount of available muscle tissue to target with stem cell, gene or drug therapy [16]. Thus, use of an appropriate model that more accurately reflects the histopathology of DMD fibrosis may better direct current research. Recent advances in exon-skipping have highlighted the importance of a suitable murine model in pre-clinical studies. In 2010, Goyenvalle demonstrated an increase in dystrophin expression in severely-affected mice lacking both utrophin and dystrophin following multiple injections with a morpholino oligomer targeted to exon 23 of the dystrophin gene [17]. Two years later, the same group modified their protocol to use an adeno-associated virus vector containing a small nuclear RNA specific to exon 23. A single treatment was sufficient to restore dystrophin in all muscles examined, including heart tissue, and dramatically increased life expectancy from 10 to 50 weeks of age [18]. These studies have been crucial in highlighting the need for inclusion of a more accurate DMD mouse model with which to assess the efficacy of therapeutic strategies.

Although utrophin-dystrophin-deficient (dko) mice were generated in 1997 [19–21], they are scarcely used in current studies. While a select number of research groups have used this severely-affected mouse model, the predominant model used in today’s research lab is still the mdx mouse. A few studies have suggested that haploinsufficiency of the utrophin gene (mdx/utrn+/-) may provide a more appropriate murine model of DMD [22, 23]. For example, it has been shown that the diaphragm and quadriceps muscles of mdx/utrn+/- mice become fibrotic as early as 3 and 6 months of age, respectively [23]. Many studies involving murine models of DMD have used mice younger than 6 months of age, thus the purpose of this study was to investigate extent of fibrosis in the mdx/utrn+/- mouse at an earlier age to determine its suitability as a more accurate representation of the human pathology. Additionally, since quadriceps and diaphragm muscles were the only groups examined in the aforementioned study, we focused our study on the gastrocnemius muscle as it has often been used as a site of cell implant [24]. In the present study, we examine extent of fibrosis and muscle regeneration in 8 week-old mice as well as aged 10 month-old mice and provide a comprehensive analysis of these parameters in various skeletal muscles that are used in DMD research.

## Materials and Methods

### Animal care and ethics statement

Experiments were performed at The Lawson Health Research Institute at St. Joseph’s Health Care (SJHC) in London, Ontario. Female C57Bl/6 mice (5–7 weeks old upon arrival) were purchased from Charles Rivers and mdx/utrn+/- mice, originally generated by Dr.’s Mark Grady and Josh Sanes (Washington University, St. Louis) [12], were generously provided to us by Dr. Robert Grange (Virginia Polytechnic and State University) and maintained in the Animal Care Facility at SJHC. Colonies were maintained under controlled conditions (19–23˚C, 12 hour light/dark cycles) and allowed water and food *ad libitum*. 7 to 8 week-old and 10 month-old male and female C57Bl/6, mdx and mdx/utrn+/- mice were used in this study. Only 7–8 week-old dko mice were used since these mice do not tend to live past 20 weeks of age (n = 3 for all groups). For comparison of various skeletal muscles, twelve week-old mdx/utrn+/- male and female mice were used. All procedures involving animal experiments were carried out in strict accordance with the Canadian Council on Animal Care (CCAC) and were approved by the Animal Use Subcommittee at Western University.

### Genotyping mdx, mdx/utrn+/- and dko mice

Genomic DNA from tail snips or ear notch tissue was used for genotyping. Presence of the utrophin gene was detected using the following set of primers (Sigma): 5’-TGCAGTGTCTCCAATAAGGTATGAAC-3’, 5’-TGCCAAGTTCTAATTCCATCAGAAGCTG -3’ (forward primers) and 5’-CTGAGTCAAACAGCTTGGAAGCCTCC-3’ (reverse primer).

### Tissue preparation and Histology

For tissue collection, mice were sacrificed via gas euthanasia. Diaphragm and gastrocnemius (GM) muscles from 8 week-old and 10 month-old mice were dissected and immediately fixed in formalin for 24–48h and embedded in paraffin. For a more comprehensive analysis of disease manifestation in mdx/utrn+/- mice, diaphragm, quadriceps, soleus, tibialis anterior (TA) and gastrocnemius (GM) muscles were similarly isolated from 12 week-old mdx/utrn+/- mice. Extreme care was taken to ensure muscles were embedded in the same orientation across each muscle group. Tissue blocks were sectioned at 5um thickness and dried in an oven at 37˚C overnight. To achieve representative sections from the whole muscle tissue, serial sections were taken every 20 slices, except for the diaphragm muscle where serial section were taken every 3 slices. Tissue sections were then deparaffinised and rehydrated in a series of xylene and ethanol washes to prepare them for subsequent Masson’s trichrome staining for collagen content, or haematoxylin and eosin staining to visualize regenerating myofibers. Serial sections were used for the two staining methods to ensure that analysis of both collagen content and muscle regeneration referred to the same samples. Following the staining step, slides were dehydrated, washed in xylene and mounted with Permount mounting medium.

### Microscopy and Image Analysis

Histological images were acquired on a Zeiss Axioscope microscope under a 20x objective using Northern Eclipse software. Non-overlapping fields of view of the entire tissue were taken for each section. Five sections were imaged per tissue. For assessment of myofiber damage, percentage of centrally-located nuclei was used as an indication of regeneration. All fields of view containing cross-sectional myofibers were imaged and manually analysed. Collagen content was assessed across the entire tissue slice and automatically quantified using an in-house colour thresholding algorithm written in MATLAB 2010a (Mathworks, Natick, MA, USA). Briefly, all images were transformed into Lab colour space allowing the isolation of the colour and lightness components of each pixel. A k-means clustering algorithm was then applied to the colour components of each individual image to partition the pixels into groups of relatively ‘red’ or ‘blue’ colour values. A uniform threshold was applied to all images to mask regions with high lightness (appearing as white). Finally, morphological closing operations were performed on the ‘red’ and ‘blue’ regions to fill any gaps less than 3 pixels in radius. The percent of collagen present in each image was calculated as the area of the remaining ‘blue’ region divided by the area of the entire image. Automatic thresholds were manually verified with labelled colour overlays on the original histology images to ensure that collagen presence was accurately identified.

### Statistical Analysis

For quantified images, a one-way ANOVA was performed followed by Tukey’s post-hoc test to determine difference between groups using GraphPad Prism. Differences between groups were considered significant at a *p*-value less than 0.05 (n = 3 for wild-type and dko mice and n = 5 for mdx and mdx/utrn+/- mice).

## Results

### Fibrosis is present at ten months of age in mdx/utrn+/-, but not mdx, gastrocnemius muscle

**Figure 1:**
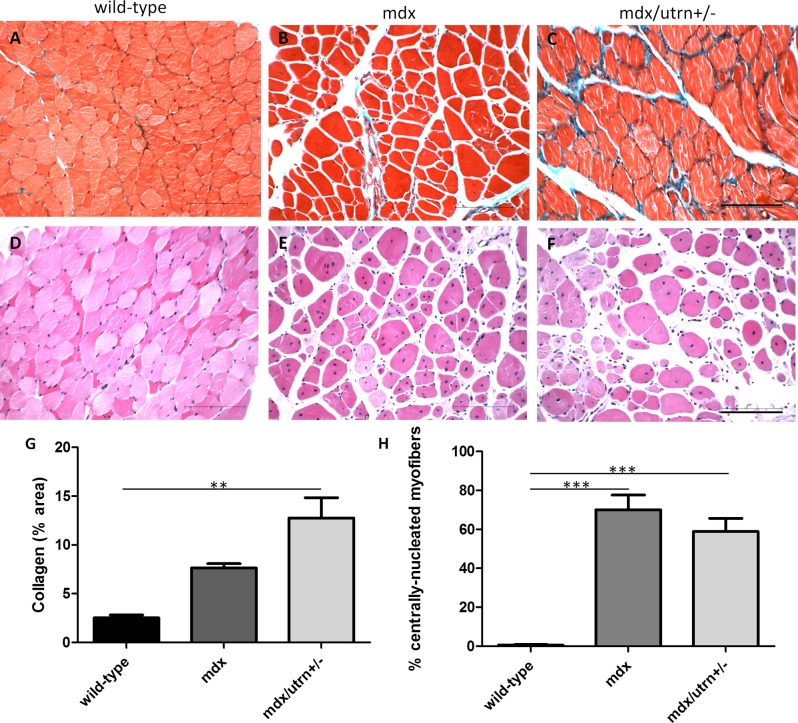
Muscle pathology in 10 month-old GM muscle of wild-type, mdx and mdx/utrn+/- mice. Extent of total collagen staining (blue) in 10 month-old wild-type (A), mdx (B) and mdx/utrn+/- (C) GM muscle was used as a marker of fibrosis. Proportion of centrally nucleated fibers in the same tissues (D, E, and F) were measured to assess extent of regeneration. Quantification of total collagen staining (G) and proportion of centrally-nucleated myofibers (H) is represented as the mean +SD. * represents p<0.05 and ** represents p<0.01 (scale bar = 100μm).

At ten months of age, mdx gastrocnemius (GM) muscle did not differ in collagen content compared to GM muscle of wild-type mice (Fig. 1A-C). In contrast, haploinsufficiency of utrophin led to significant collagen deposition in GM tissue of aged mdx/utrn+/- mice (mean±SD: 12.76%±3.06, p = 0.0033). There was no significant difference in collagen deposition between mdx and mdx/utrn+/- GM muscle. Although GM muscles of aged mdx mice did not show significant fibrosis compared to healthy wild-type controls, there was a large proportion of centrally-located nuclei in the myofibers indicative of muscle regeneration in both mdx (70.06%±7.6) and mdx/utrn+/- (58.86%±6.7) mice, respectively (p<0.0001, Fig. 1D-F).

### DMD-associated fibrosis is present in both aged mdx and mdx/utrn+/- diaphragm muscle

**Figure 2:**
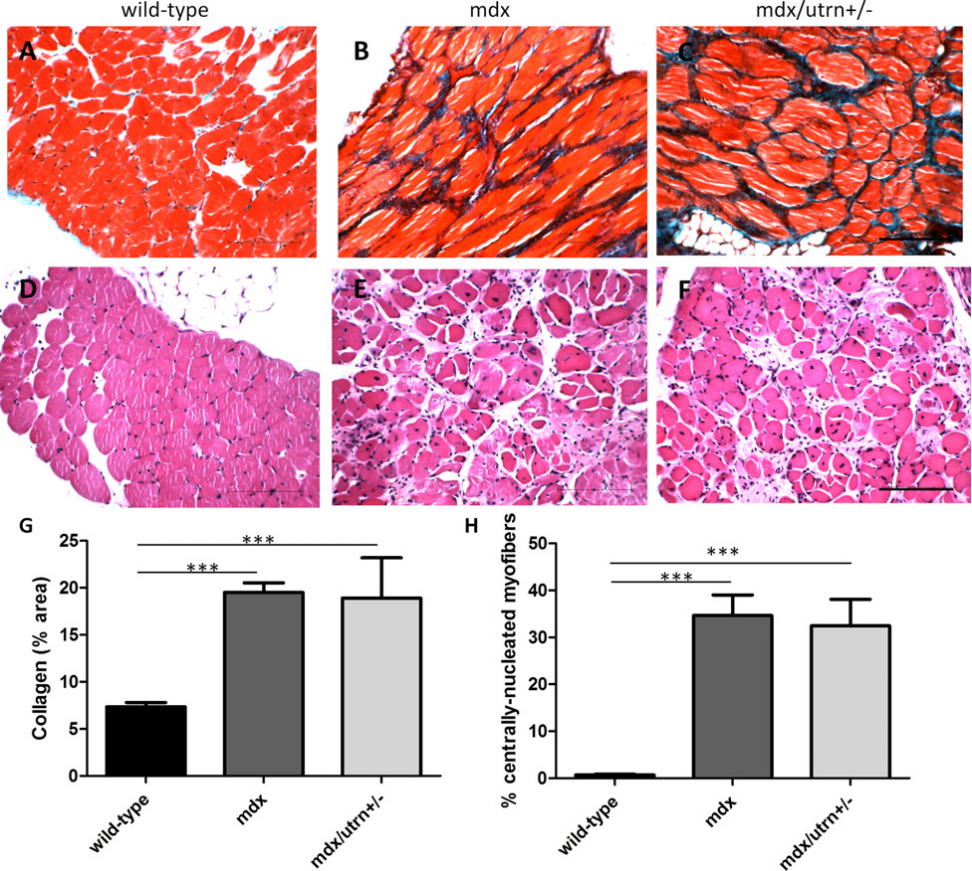
Muscle pathology in 10 month-old diaphragm muscle of wild-type, mdx and mdx/utrn+/- mice. Extent of total collagen staining (blue) in 10 month-old wild-type (A), mdx (B) and mdx/utrn+/- (C) diaphragms was used as a marker of fibrosis. Proportion of centrally nucleated fibers in the same tissues (D, E, and F) were measured to assess extent of regeneration. Quantification of total collagen staining (G) and proportion of centrally-nucleated myofibers (H) is represented as the mean +SD. * represents p<0.05 and *** represents p<0.001 (scale bar = 100μm).

Both mdx and mdx/utrn+/- diaphragm muscles were significantly fibrotic in ten month-old mice compared to age-matched wild-type controls (Fig. 2A-C, p = 0.026), indicated by an approximate 2.5 fold increase in collagen content. Both mdx and mdx/utrn+/- diaphragm muscle revealed a significantly higher percent of centrally-located nuclei (34.65%±4.4 and 32.45%±5.6, respectively) compared to the age-matched wild-type mice (p<0.0001). There was no significant difference in number of centrally-nucleated myofibers between mdx and mdx/utrn+/- mice (Fig. 2D-F).

### Young mdx/utrn+/- and dko, but not mdx, gastrocnemius muscle mimics DMD histopathology

**Figure 3:**
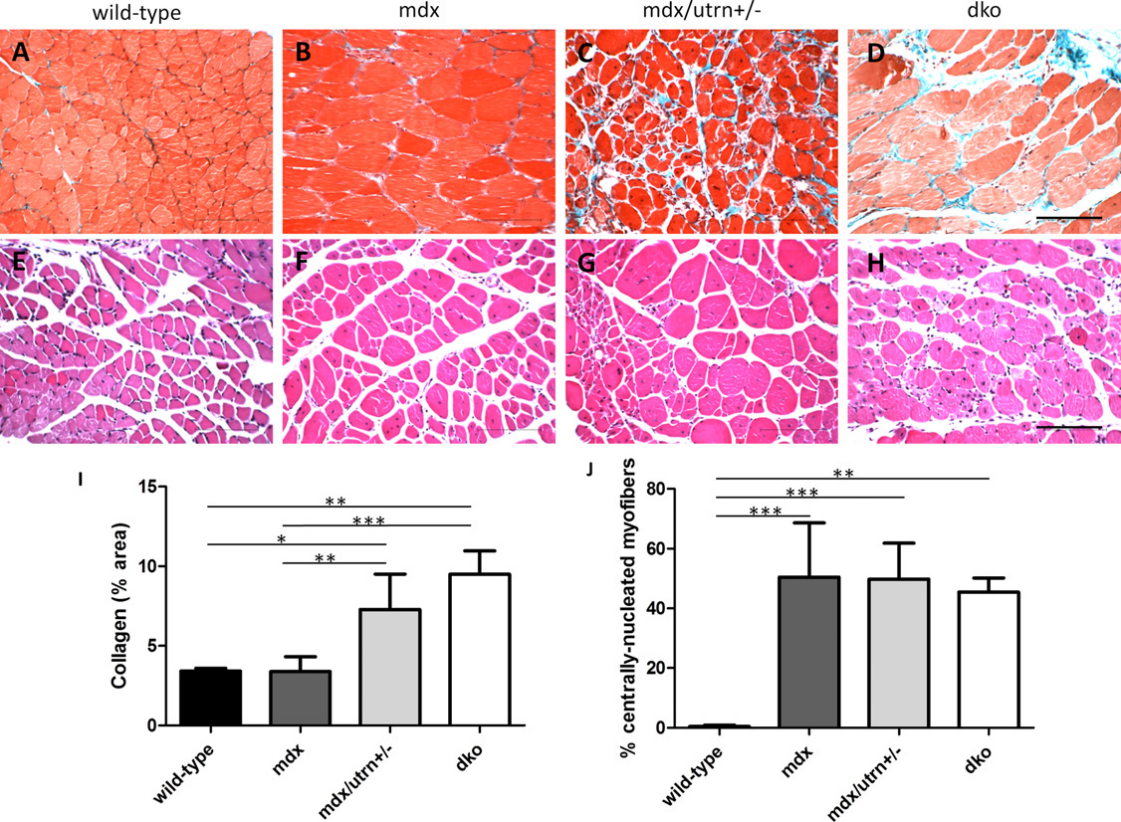
Muscle pathology in 8 week-old GM muscle of wild-type, mdx, mdx/utrn+/- and dko mice. Extent of total collagen staining (blue) in 8 week-old wild-type (A), mdx (B), mdx/utrn+/- (C) and dko (D) GM muscles was used as a marker of fibrosis. Proportion of centrally nucleated fibers in the same tissues (E, F, G, and H) were measured to assess extent of regeneration. Quantification of total collagen staining (I) and proportion of centrally-nucleated myofibers (J) is represented as the mean +SD. * represents p<0.05, ** represents p<0.01, and *** represents p<0.001 (scale bar = 100μm).

Fibrosis was assessed in 8 week-old GM muscle of wild-type, mdx, mdx/utrn+/- and dko mice (Fig. 3A-D). There was no observed fibrosis in mdx GM (3.38%±0.9) compared to wild-type GM (3.40 ±0.2). There was a significantly higher amount of collagen deposition in mdx/utrn+/- GM (7.28%±2.2) compared to healthy wild-type and mdx tissue; however this difference was not significant between GM muscles of dko mice (9.49%±1.5). Overall, quantification of collagen content indicates that fibrosis is absent in wild-type and mdx GM muscle, but present in mdx/utrn+/- and dko GM muscle at a young age (p = 0.0003). Muscle regeneration was significantly higher in all three murine models of DMD compared to the wild-type controls (Fig. 3E-H). The proportion of centrally-located nuclei did not differ, however, between mdx (50.41%±18.2), mdx/utrn+/- (49.78%±12.0) and dko GM muscle (45.44%±4.7, p = 0.0007).

### Fibrosis is present in diaphragm muscle at a young age in all three murine models of DMD

**Figure 4:**
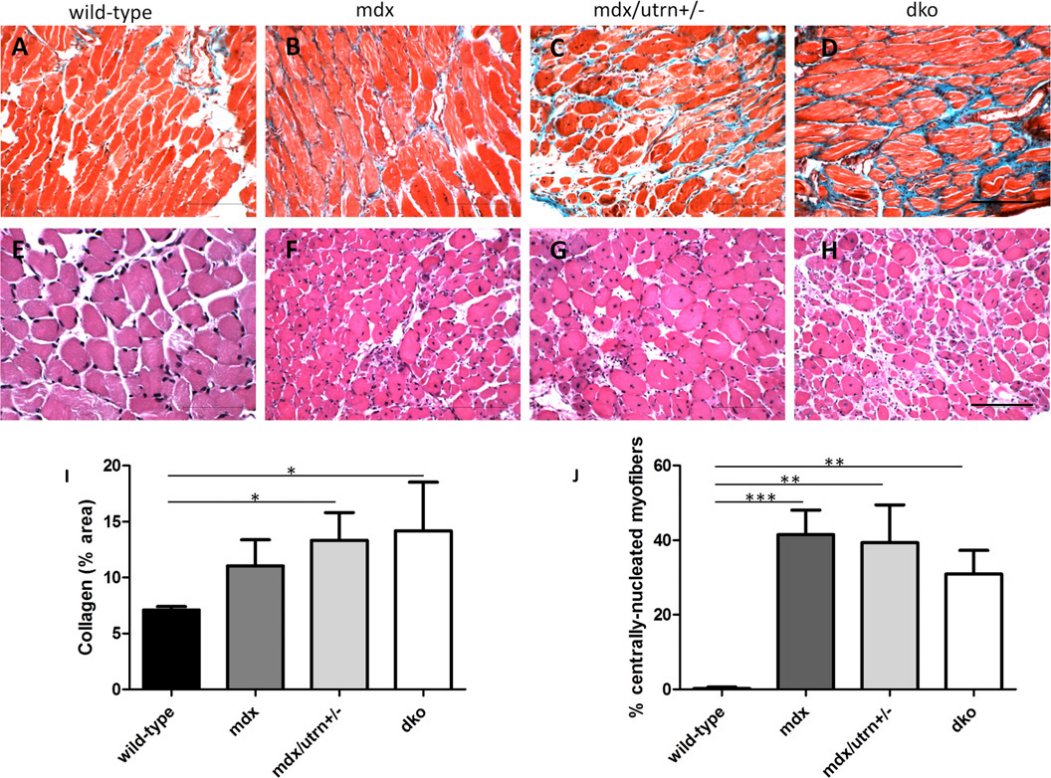
Muscle pathology in 8 week-old diaphragm muscle of wild-type, mdx, mdx/utrn+/- and dko mice. Extent of total collagen staining (blue) in 8 week-old wild-type (A), mdx (B), mdx/utrn+/- (C) and dko (D) diaphragms was used as a marker of fibrosis. Proportion of centrally nucleated fibers in the same tissues (E, F, G, and H) were measured to assess extent of regeneration. Quantification of total collagen staining (I) and proportion of centrally-nucleated myofibers (J) is represented as the mean +SD. * represents p<0.05, ** represents p<0.01, and *** represents p<0.001 (scale bar = 100μm)

Collagen content in the diaphragm muscle was not determined to be significantly different between mdx mice (11.04%±2.3) and the two more severely-affected animal models or between healthy wild-type and mdx mice (Fig. 4A-D). Collagen deposition was significantly higher in the diaphragm of both dko (14.17%±4.4) and mdx/utrn+/- (13.32%±2.5) mice at 8 weeks of age compared to age-matched wild-type diaphragm muscle (7.1%±0.3, p = 0.0235). Myofiber regeneration was significantly higher in the diaphragm muscles of mdx (41.48%±6.6) mdx/utrn+/- (39.39%±10.18) and dko (30.95%±6.4) mice compared to diaphragm muscle in wild-type mice (Fig. 4E-H, p<0.0001).

### Extent of fibrosis differs between muscle groups in the mdx/utrn+/- mouse

**Figure 5:**
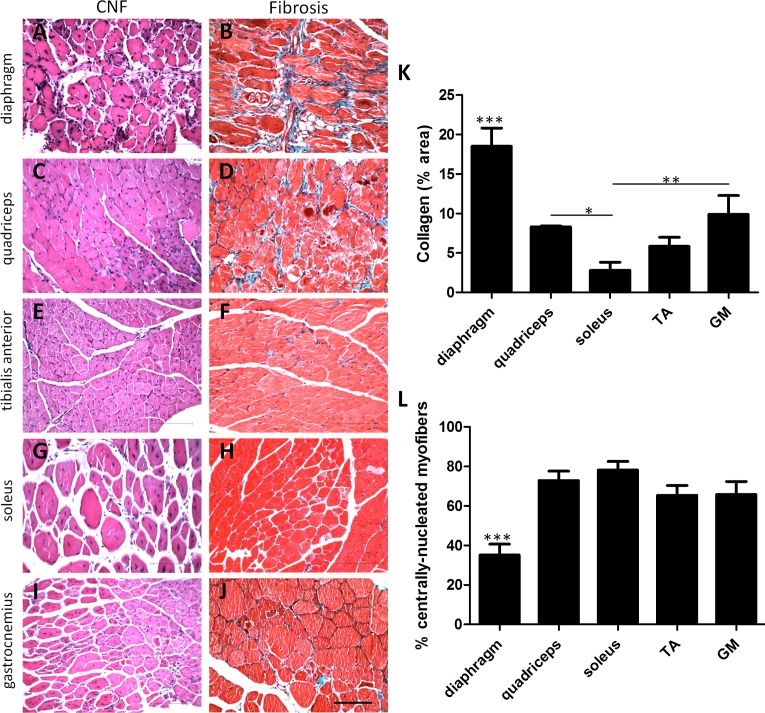
Comparison of histopathology of 12 week-old mdx/utrn+/- diaphragm, quadriceps, soleus, tibialis anterior and gastrocnemius muscles. Extent of collagen staining (A,C,E,G,I) and muscle regeneration (B,D,F,H,J) in 12 week-old mdx/utrn+/- mice. Quantification of total collagen staining (K) and centrally-nucleated myofibers (CNF, L) is represented as the mean ±SD. * represents p<0.05, ** represents p<0.01, and *** represents p<0.001 (scale bar = 100μm). TA = tibialis anterior, GM = gastrocnemius.

Diaphragm, quadriceps, soleus, tibialis anterior (TA) and gastrocnemius (GM) muscles were analyzed from 12 week-old mdx/utrn+/- mice (Fig. 5). Collagen content in diaphragm muscle was significantly higher than all other muscles assessed (p<0.0001). Both the quadriceps (8.31%±0.1) and GM muscles (9.89%±2.4) were higher in collagen content than the soleus muscle (2.80%±1.0) at this age. Extent of fibrosis was not significantly different in the TA muscle (5.85%±1.1) compared to the quadriceps, GM or soleus muscles. Myofiber regeneration, indicated by centrally-located nuclei, was significantly higher in all four lower limb muscles compared to myofibers in the diaphragm, which only had 35.1% of myofibers in a regenerative state (p<0.0001).

## Discussion

Fibrosis is a hallmark feature of Duchenne muscular dystrophy (DMD), yet the most widely- used murine model for the disease, the mdx mouse, does not model this aspect of DMD until and advanced age [25]. Consistent with previous reports [26], this study demonstrates that while the diaphragm muscle becomes fibrotic in aged mdx mice hind limb muscle does not. Many studies that investigate the potential use of stem cell or gene therapy for DMD use hind limb muscles, such as the gastrocnemius muscle (GM), for sites of injection [27]. Here, we demonstrate that neither young nor aged mdx mice develop a significant amount of fibrosis in the GM muscle compared to healthy wild-type controls of the same age. The fact that mdx mice lack this excessive deposition of extracellular matrix proteins may lead researchers to overestimate treatment efficacy in this murine model since there is a lack of environmental hostility when testing their therapeutic agents. Alternatively, the mdx/utrn+/- mouse exhibits hind limb fibrosis at 10 months of age. Interestingly, a large proportion of regenerating myofibers, indicative of damage, was measured in both aged animal models. Centrally-located nuclei are a hallmark of muscle regeneration, and are often used as an indicator of muscle pathogenesis [28]. However, as we have clearly demonstrated in this study, the presence of centric nuclei alone does not provide an accurate measure of the degree of muscle degeneration. Indeed, while centric nuclei are prominent in the mdx mouse model of DMD, other aspects of the disease in humans, particularly fibrosis, are not phenocopied. This may be due, at least in part, to the upregulation of utrophin in the mdx mouse. As a result, overall muscle damage is significantly less in mdx mice than in utrophin heterozygotes and double knockout animals. Interestingly, we have further shown that in muscle tissues exhibiting higher levels of collagen (eg diaphragm), proportions of centric nuclei are lower. In contrast, the GM muscle in mdx mouse exhibits a significantly higher number of centric nuclei, but little deposition of collagen. These findings are consistent with earlier reports hypothesizing that cenrally-nucleated myofibers are more resistant to mechanical stress, which may in part explain for the differences in the pathology observed in diaphragm versus hind limb skeletal muscles [29]

Since DMD manifests at a young age in humans and because most DMD research is performed in younger animals, we also investigated extent of fibrosis in eight week-old mice. Although the GM muscles in young mdx mice are in a regenerative state as evidenced by the presence of centrally-located nuclei, we did not measure a significant degree of fibrosis in them. In comparison, there was a higher level of collagen measured in both moderately-affected mdx/utrn+/- and severely-affected dko mice relative to their healthy counterparts, suggesting fibrotic progression in these animals. Given the discrepancy between these findings and those of Zhou et al. (2008) who reported that collagen deposition was comparable in the quadriceps muscle of both mdx and mdx:utrn+/- mice at three months of age, we also characterized the extent of fibrosis in a number of skeletal muscles isolated from the utrophin heterozygote mouse at this age. As expected, the diaphragm muscle was highly fibrotic compared to the quadriceps, soleus, tibialis anterior and GM muscles. In comparison, there was no detectable difference in collagen deposition between the quadriceps, TA or GM muscles; this is an important finding considering that all three muscles are used in current research and therefore should display signs of fibrosis [30, 31]. Interestingly, we measured a lower amount of collagen content in the slow-twitch soleus muscle than the fast-twitch quadriceps and GM muscles. The fact that fibrotic progression is not equal between individual skeletal muscles is an important factor to consider in future studies.

Taken together, this study supports previous literature that argues for the replacement of the mdx mouse with more severely-affected models of DMD, such as the mdx/utrn+/- or dko mouse [19, 20]. Although dko mice develop debilitating fibrosis at a young age, these animals do not tend to live past twenty weeks and thus are not generally used in studies examining the long term efficacy of a therapeutic intervention. Similarly, although the diaphragm muscle in all three murine models develops fibrosis at some point, a more accurate model of DMD should reflect disease manifestation in axial limbs as well. Nevertheless, it would be optimal for any therapeutic drug under investigation to show efficacy in the diaphragm muscle since failure of this respiratory organ is a common cause of death in DMD patients [32, 33]. Since the mdx/utrn+/- mouse develops fibrosis in both hind limb and respiratory skeletal muscles at a young age while not being so affected such that it dies prematurely, we provide further evidence here that it may be an appropriate and useful model of DMD. Furthermore, we report here that this increased disease severity in the mdx/utrn+/- mouse compared to its mdx counterpart is apparent by two months of age. The mdx/utrn+/- mouse is also a suitable precursor model for scaling studies to large animal models of DMD such as the golden retriever model (GRMD), which exhibits signs of increased endomysial fibrosis as early as 15 days after birth, with severe fibrosis by 9 months of age [34–36].

## Conclusion

In addition to the degeneration of muscle in DMD patients, fibrosis is a prominent and debilitating aspect of the disease. Use of an animal model that accurately reflects both of these features will be absolutely integral to the development of treatment strategies to not only increase quality of life but also slow the progression of the disease and ultimately increase life expectancy.
